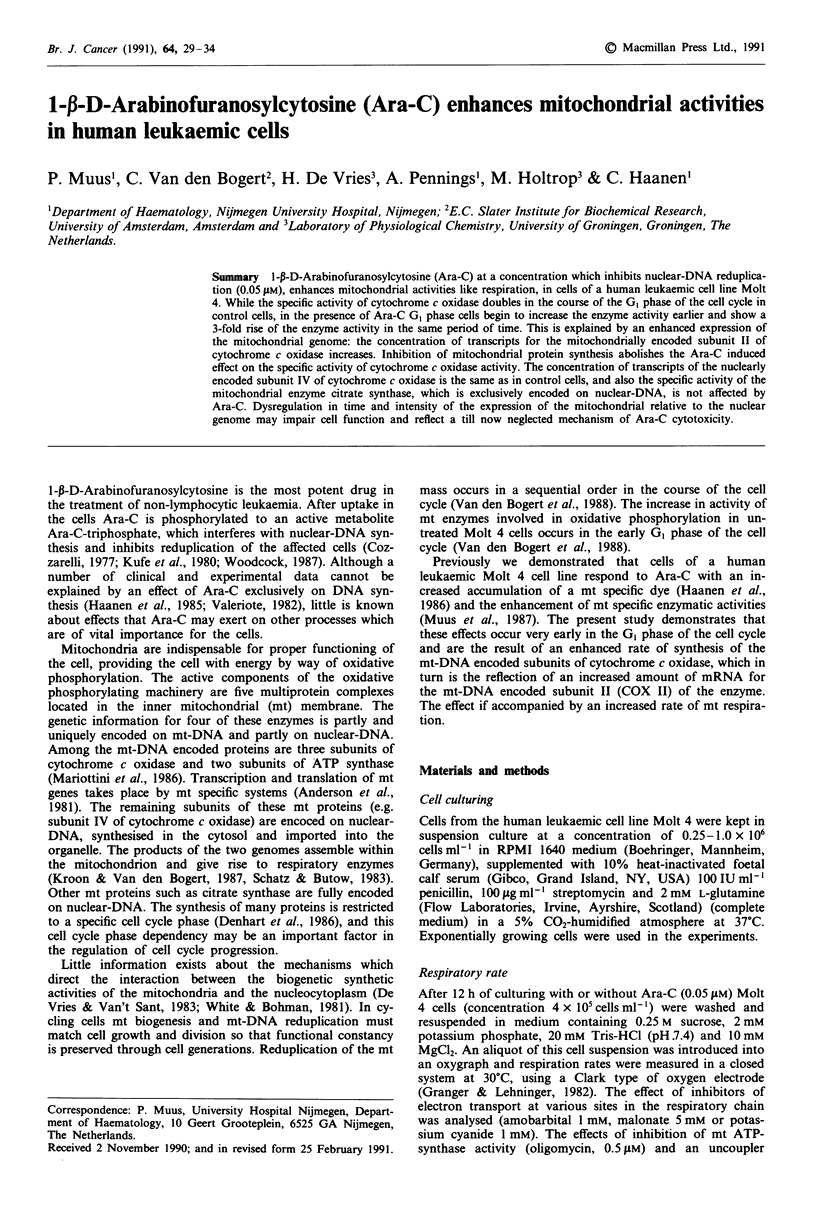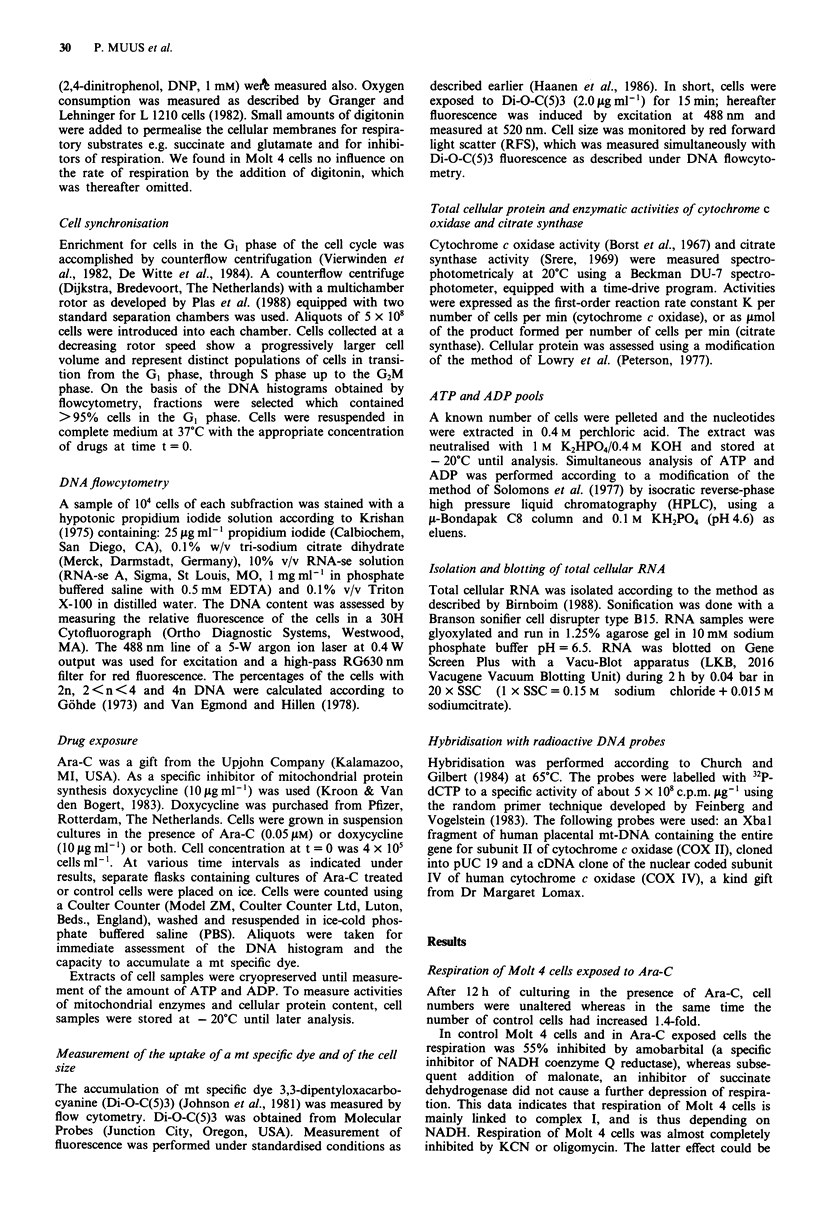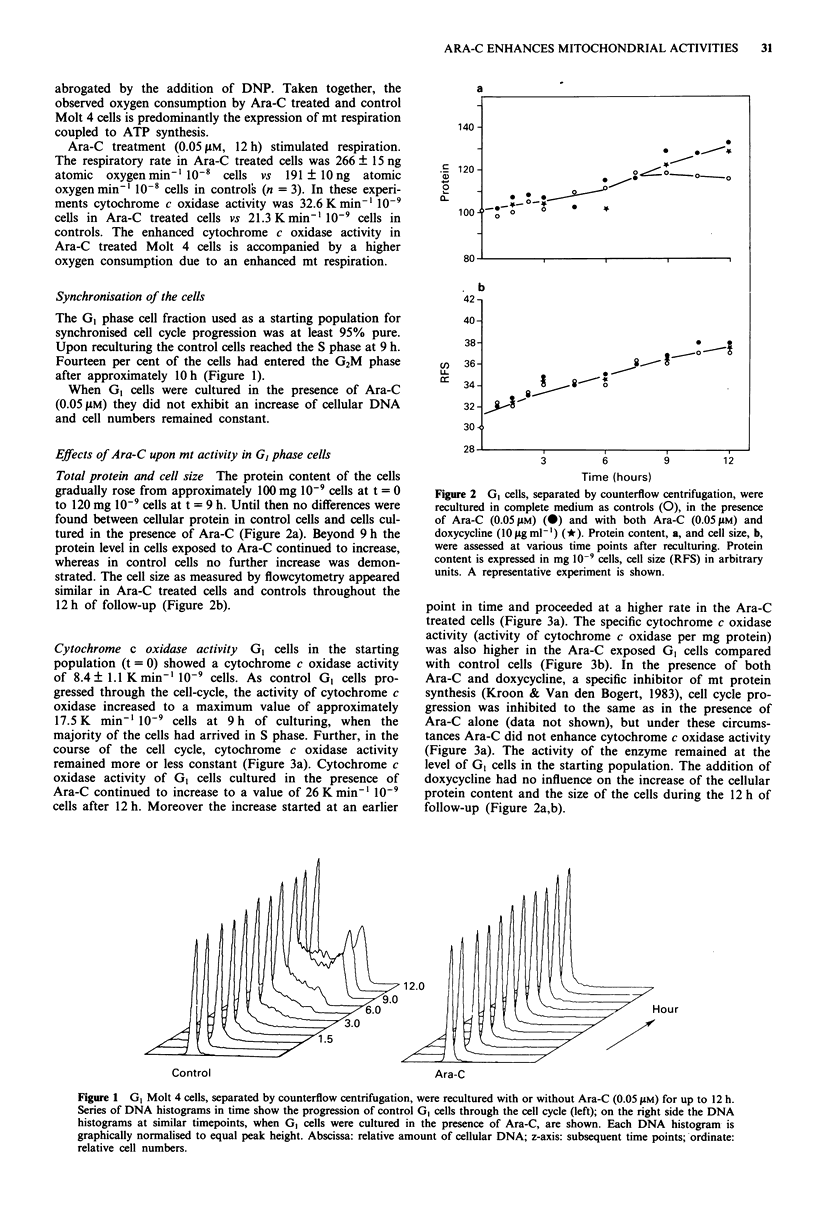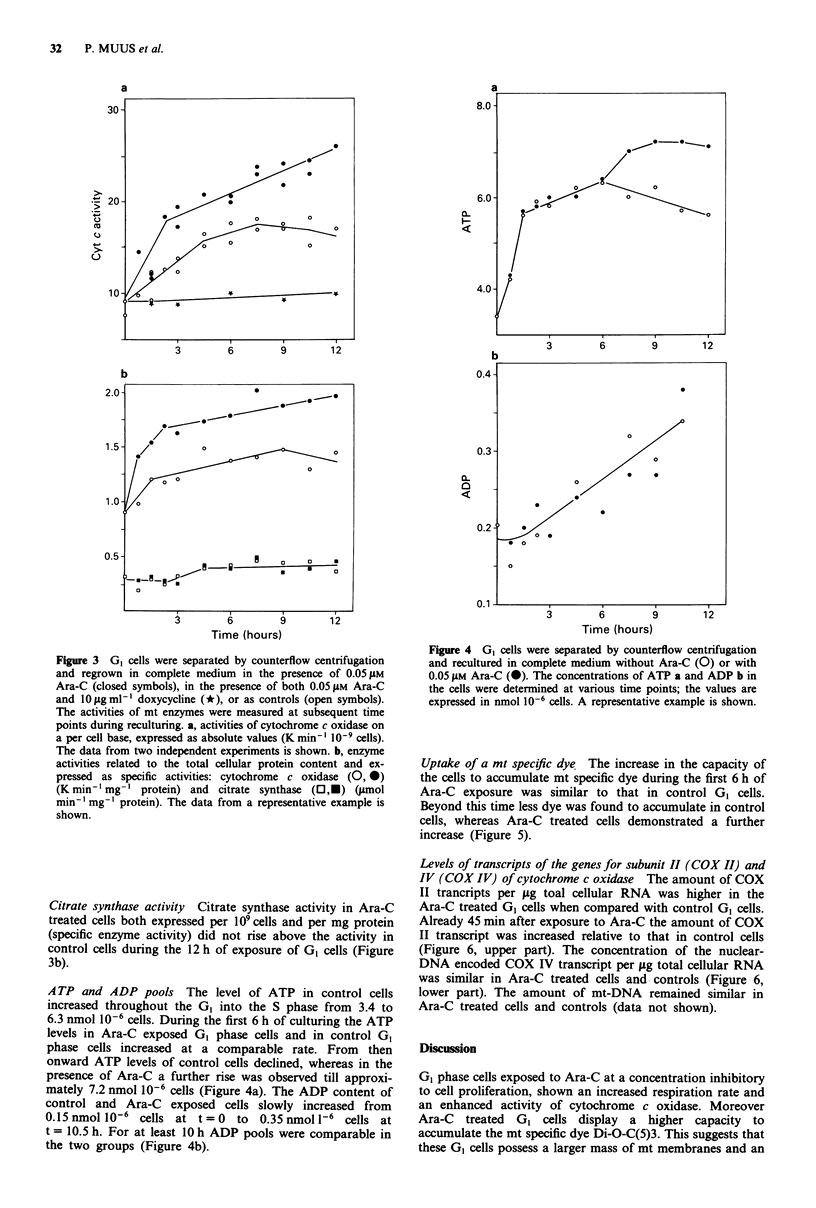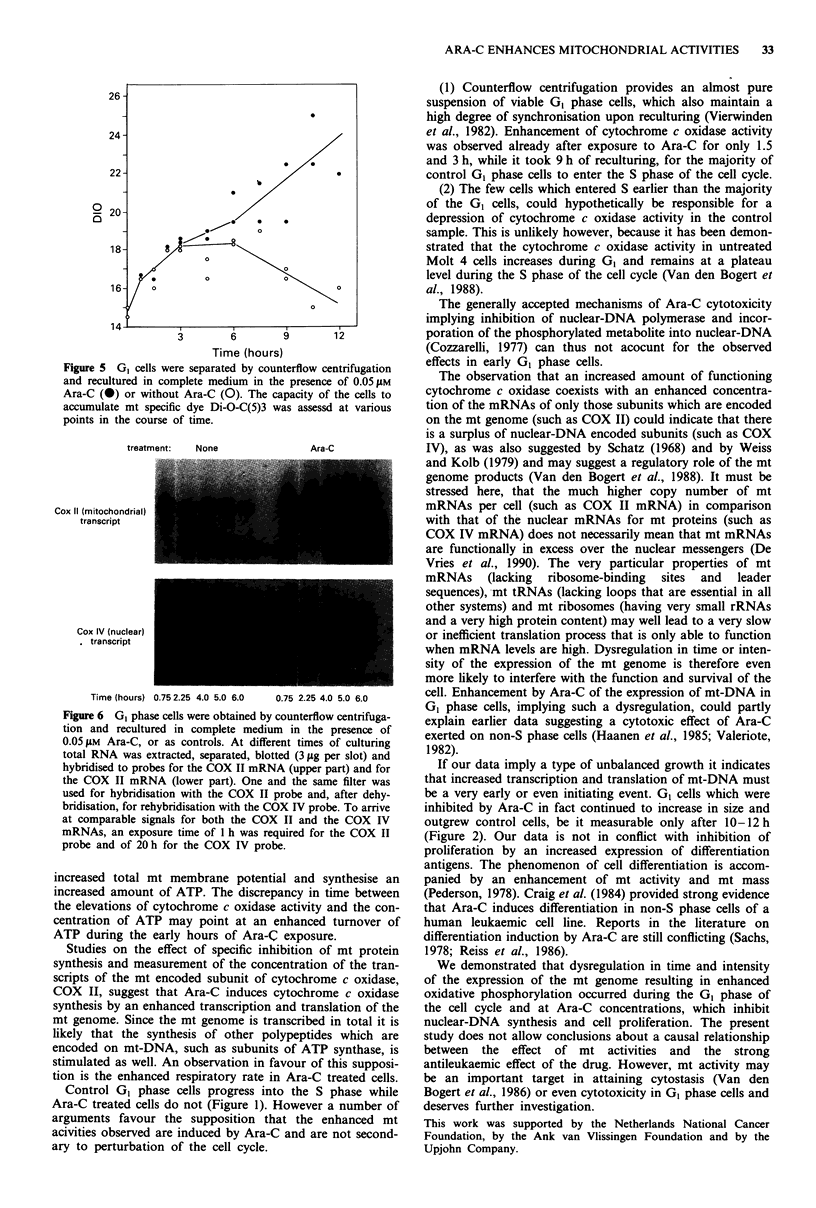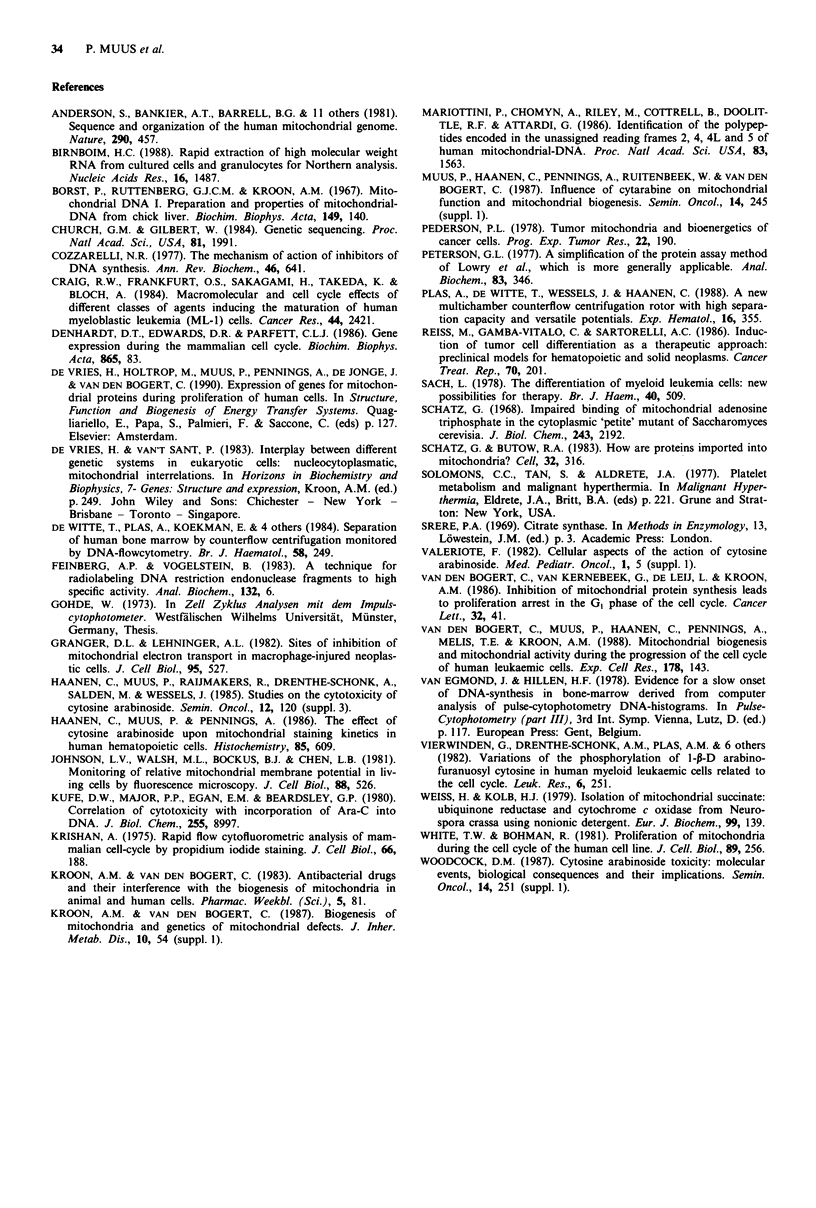# 1-beta-D-arabinofuranosylcytosine (Ara-C) enhances mitochondrial activities in human leukaemic cells.

**DOI:** 10.1038/bjc.1991.234

**Published:** 1991-07

**Authors:** P. Muus, C. Van den Bogert, H. De Vries, A. Pennings, M. Holtrop, C. Haanen

**Affiliations:** Department of Haematology, Nijmegen University Hospital, The Netherlands.

## Abstract

**Images:**


Br. J. Cancer (1991), 64, 29-34 © Macmillan Press Ltd., 1991

### 1-β-D-Arabinofuranosylcytosine (Ara-C) enhances mitochondrial activities in human leukaemic cells

P. Muus[1], C. Van den Bogert[2], H. De Vries[3], A. Pennings[1], M. Holtrop[3] & C. Haanen[1]

[1]*Department of Haematology, Nijmegen University Hospital, Nijmegen;* [2]*E.C. Slater Institute for Biochemical Research, University of Amsterdam, Amsterdam and* [3]*Laboratory of Physiological Chemistry, University of Groningen, Groningen, The Netherlands.*

**Summary** 1-β-D-Arabinofuranosylcytosine (Ara-C) at a concentration which inhibits nuclear-DNA reduplication (0.05 μM), enhances mitochondrial activities like respiration, in cells of a human leukaemic cell line Molt 4. While the specific activity of cytochrome *c* oxidase doubles in the course of the $G_1$ phase of the cell cycle in control cells, in the presence of Ara-C $G_1$ phase cells begin to increase the enzyme activity earlier and show a 3-fold rise of the enzyme activity in the same period of time. This is explained by an enhanced expression of the mitochondrial genome: the concentration of transcripts for the mitochondrially encoded subunit II of cytochrome *c* oxidase increases. Inhibition of mitochondrial protein synthesis abolishes the Ara-C induced effect on the specific activity of cytochrome *c* oxidase activity. The concentration of transcripts of the nuclearly encoded subunit IV of cytochrome *c* oxidase is the same as in control cells, and also the specific activity of the mitochondrial enzyme citrate synthase, which is exclusively encoded on nuclear-DNA, is not affected by Ara-C. Dysregulation in time and intensity of the expression of the mitochondrial relative to the nuclear genome may impair cell function and reflect a till now neglected mechanism of Ara-C cytotoxicity.

1-β-D-Arabinofuranosylcytosine is the most potent drug in the treatment of non-lymphocytic leukaemia. After uptake in the cells Ara-C is phosphorylated to an active metabolite Ara-C-triphosphate, which interferes with nuclear-DNA synthesis and inhibits reduplication of the affected cells (Cozzarelli, 1977; Kufe *et al.*, 1980; Woodcock, 1987). Although a number of clinical and experimental data cannot be explained by an effect of Ara-C exclusively on DNA synthesis (Haanen *et al.*, 1985; Valeriote, 1982), little is known about effects that Ara-C may exert on other processes which are of vital importance for the cells.

Mitochondria are indispensable for proper functioning of the cell, providing the cell with energy by way of oxidative phosphorylation. The active components of the oxidative phosphorylating machinery are five multiprotein complexes located in the inner mitochondrial (mt) membrane. The genetic information for four of these enzymes is partly and uniquely encoded on mt-DNA and partly on nuclear-DNA. Among the mt-DNA encoded proteins are three subunits of cytochrome *c* oxidase and two subunits of ATP synthase (Mariottini *et al.*, 1986). Transcription and translation of mt genes takes place by mt specific systems (Anderson *et al.*, 1981). The remaining subunits of these mt proteins (e.g. subunit IV of cytochrome *c* oxidase) are encoced on nuclear-DNA, synthesised in the cytosol and imported into the organelle. The products of the two genomes assemble within the mitochondrion and give rise to respiratory enzymes (Kroon & Van den Bogert, 1987, Schatz & Butow, 1983). Other mt proteins such as citrate synthase are fully encoded on nuclear-DNA. The synthesis of many proteins is restricted to a specific cell cycle phase (Denhart *et al.*, 1986), and this cell cycle phase dependency may be an important factor in the regulation of cell cycle progression.

Little information exists about the mechanisms which direct the interaction between the biogenetic synthetic activities of the mitochondria and the nucleocytoplasm (De Vries & Van't Sant, 1983; White & Bohman, 1981). In cycling cells mt biogenesis and mt-DNA reduplication must match cell growth and division so that functional constancy is preserved through cell generations. Reduplication of the mt mass occurs in a sequential order in the course of the cell cycle (Van den Bogert *et al.*, 1988). The increase in activity of mt enzymes involved in oxidative phosphorylation in untreated Molt 4 cells occurs in the early $G_1$ phase of the cell cycle (Van den Bogert *et al.*, 1988).

Previously we demonstrated that cells of a human leukaemic Molt 4 cell line respond to Ara-C with an increased accumulation of a mt specific dye (Haanen *et al.*, 1986) and the enhancement of mt specific enzymatic activities (Muus *et al.*, 1987). The present study demonstrates that these effects occur very early in the $G_1$ phase of the cell cycle and are the result of an enhanced rate of synthesis of the mt-DNA encoded subunits of cytochrome *c* oxidase, which in turn is the reflection of an increased amount of mRNA for the mt-DNA encoded subunit II (COX II) of the enzyme. The effect if accompanied by an increased rate of mt respiration.

### Materials and methods

### Cell culturing

Cells from the human leukaemic cell line Molt 4 were kept in suspension culture at a concentration of $0.25-1.0 \times 10^6$ cells $ml^{-1}$ in RPMI 1640 medium (Boehringer, Mannheim, Germany), supplemented with 10% heat-inactivated foetal calf serum (Gibco, Grand Island, NY, USA) 100 IU $ml^{-1}$ penicillin, 100 μg $ml^{-1}$ streptomycin and 2 mM L-glutamine (Flow Laboratories, Irvine, Ayrshire, Scotland) (complete medium) in a 5% $CO_2$-humidified atmosphere at 37°C. Exponentially growing cells were used in the experiments.

### Respiratory rate

After 12 h of culturing with or without Ara-C (0.05 μM) Molt 4 cells (concentration $4 \times 10^5$ cells $ml^{-1}$) were washed and resuspended in medium containing 0.25 M sucrose, 2 mM potassium phosphate, 20 mM Tris-HCl (pH 7.4) and 10 mM $MgCl_2$. An aliquot of this cell suspension was introduced into an oxygraph and respiration rates were measured in a closed system at 30°C, using a Clark type of oxygen electrode (Granger & Lehninger, 1982). The effect of inhibitors of electron transport at various sites in the respiratory chain was analysed (amobarbital 1 mM, malonate 5 mM or potassium cyanide 1 mM). The effects of inhibition of mt ATP-synthase activity (oligomycin, 0.5 μM) and an uncoupler

Correspondence: P. Muus, University Hospital Nijmegen, Department of Haematology, 10 Geert Grooteplein, 6525 GA Nijmegen, The Netherlands.
Received 2 November 1990; and in revised form 25 February 1991.

(2,4-dinitrophenol, DNP, 1 mM) were measured also. Oxygen consumption was measured as described by Granger and Lehninger for L 1210 cells (1982). Small amounts of digitonin were added to permealise the cellular membranes for respiratory substrates e.g. succinate and glutamate and for inhibitors of respiration. We found in Molt 4 cells no influence on the rate of respiration by the addition of digitonin, which was thereafter omitted.

### *Cell synchronisation*

Enrichment for cells in the $G_1$ phase of the cell cycle was accomplished by counterflow centrifugation (Vierwinden *et al.*, 1982, De Witte *et al.*, 1984). A counterflow centrifuge (Dijkstra, Bredevoort, The Netherlands) with a multichamber rotor as developed by Plas *et al.* (1988) equipped with two standard separation chambers was used. Aliquots of $5 \times 10^8$ cells were introduced into each chamber. Cells collected at a decreasing rotor speed show a progressively larger cell volume and represent distinct populations of cells in transition from the $G_1$ phase, through S phase up to the $G_2M$ phase. On the basis of the DNA histograms obtained by flowcytometry, fractions were selected which contained >95% cells in the $G_1$ phase. Cells were resuspended in complete medium at 37°C with the appropriate concentration of drugs at time $t = 0$.

### *DNA flowcytometry*

A sample of $10^4$ cells of each subfraction was stained with a hypotonic propidium iodide solution according to Krishan (1975) containing: 25 $\mu g\ ml^{-1}$ propidium iodide (Calbiochem, San Diego, CA), 0.1% w/v tri-sodium citrate dihydrate (Merck, Darmstadt, Germany), 10% v/v RNA-se solution (RNA-se A, Sigma, St Louis, MO, 1 $mg\ ml^{-1}$ in phosphate buffered saline with 0.5 mM EDTA) and 0.1% v/v Triton X-100 in distilled water. The DNA content was assessed by measuring the relative fluorescence of the cells in a 30H Cytofluorograph (Ortho Diagnostic Systems, Westwood, MA). The 488 nm line of a 5-W argon ion laser at 0.4 W output was used for excitation and a high-pass RG630 nm filter for red fluorescence. The percentages of the cells with 2n, $2 < n < 4$ and 4n DNA were calculated according to Göhde (1973) and Van Egmond and Hillen (1978).

### *Drug exposure*

Ara-C was a gift from the Upjohn Company (Kalamazoo, MI, USA). As a specific inhibitor of mitochondrial protein synthesis doxycycline (10 $\mu g\ ml^{-1}$) was used (Kroon & Van den Bogert, 1983). Doxycycline was purchased from Pfizer, Rotterdam, The Netherlands. Cells were grown in suspension cultures in the presence of Ara-C (0.05 $\mu$M) or doxycycline (10 $\mu g\ ml^{-1}$) or both. Cell concentration at $t = 0$ was $4 \times 10^5$ cells $ml^{-1}$. At various time intervals as indicated under results, separate flasks containing cultures of Ara-C treated or control cells were placed on ice. Cells were counted using a Coulter Counter (Model ZM, Coulter Counter Ltd, Luton, Beds., England), washed and resuspended in ice-cold phosphate buffered saline (PBS). Aliquots were taken for immediate assessment of the DNA histogram and the capacity to accumulate a mt specific dye.

Extracts of cell samples were cryopreserved until measurement of the amount of ATP and ADP. To measure activities of mitochondrial enzymes and cellular protein content, cell samples were stored at − 20°C until later analysis.

### *Measurement of the uptake of a mt specific dye and of the cell size*

The accumulation of mt specific dye 3,3-dipentyloxacarbocyanine (Di-O-C(5)3) (Johnson *et al.*, 1981) was measured by flow cytometry. Di-O-C(5)3 was obtained from Molecular Probes (Junction City, Oregon, USA). Measurement of fluorescence was performed under standardised conditions as described earlier (Haanen *et al.*, 1986). In short, cells were exposed to Di-O-C(5)3 (2.0 $\mu g\ ml^{-1}$) for 15 min; hereafter fluorescence was induced by excitation at 488 nm and measured at 520 nm. Cell size was monitored by red forward light scatter (RFS), which was measured simultaneously with Di-O-C(5)3 fluorescence as described under DNA flowcytometry.

### *Total cellular protein and enzymatic activities of cytochrome* c *oxidase and citrate synthase*

Cytochrome *c* oxidase activity (Borst *et al.*, 1967) and citrate synthase activity (Srere, 1969) were measured spectrophotometricaly at 20°C using a Beckman DU-7 spectrophotometer, equipped with a time-drive program. Activities were expressed as the first-order reaction rate constant K per number of cells per min (cytochrome *c* oxidase), or as $\mu$mol of the product formed per number of cells per min (citrate synthase). Cellular protein was assessed using a modification of the method of Lowry *et al.* (Peterson, 1977).

### *ATP and ADP pools*

A known number of cells were pelleted and the nucleotides were extracted in 0.4 M perchloric acid. The extract was neutralised with 1 M $K_2HPO_4$/0.4 M KOH and stored at − 20°C until analysis. Simultaneous analysis of ATP and ADP was performed according to a modification of the method of Solomons *et al.* (1977) by isocratic reverse-phase high pressure liquid chromatography (HPLC), using a $\mu$-Bondapak C8 column and 0.1 M $KH_2PO_4$ (pH 4.6) as eluens.

### *Isolation and blotting of total cellular RNA*

Total cellular RNA was isolated according to the method as described by Birnboim (1988). Sonification was done with a Branson sonifier cell disrupter type B15. RNA samples were glyoxylated and run in 1.25% agarose gel in 10 mM sodium phosphate buffer pH = 6.5. RNA was blotted on Gene Screen Plus with a Vacu-Blot apparatus (LKB, 2016 Vacugene Vacuum Blotting Unit) during 2 h by 0.04 bar in 20 × SSC (1 × SSC = 0.15 M sodium chloride + 0.015 M sodiumcitrate).

### *Hybridisation with radioactive DNA probes*

Hybridisation was performed according to Church and Gilbert (1984) at 65°C. The probes were labelled with $^{32}$P-dCTP to a specific activity of about $5 \times 10^8$ c.p.m. $\mu g^{-1}$ using the random primer technique developed by Feinberg and Vogelstein (1983). The following probes were used: an XbaI fragment of human placental mt-DNA containing the entire gene for subunit II of cytochrome *c* oxidase (COX II), cloned into pUC 19 and a cDNA clone of the nuclear coded subunit IV of human cytochrome *c* oxidase (COX IV), a kind gift from Dr Margaret Lomax.

### Results

### *Respiration of Molt 4 cells exposed to Ara-C*

After 12 h of culturing in the presence of Ara-C, cell numbers were unaltered whereas in the same time the number of control cells had increased 1.4-fold.

In control Molt 4 cells and in Ara-C exposed cells the respiration was 55% inhibited by amobarbital (a specific inhibitor of NADH coenzyme Q reductase), whereas subsequent addition of malonate, an inhibitor of succinate dehydrogenase did not cause a further depression of respiration. This data indicates that respiration of Molt 4 cells is mainly linked to complex I, and is thus depending on NADH. Respiration of Molt 4 cells was almost completely inhibited by KCN or oligomycin. The latter effect could be abrogated by the addition of DNP. Taken together, the observed oxygen consumption by Ara-C treated and control Molt 4 cells is predominantly the expression of mt respiration coupled to ATP synthesis.

Ara-C treatment (0.05 μM, 12 h) stimulated respiration. The respiratory rate in Ara-C treated cells was 266 ± 15 ng atomic oxygen min$^{-1}$ $10^{-8}$ cells *vs* 191 ± 10 ng atomic oxygen min$^{-1}$ $10^{-8}$ cells in controls (*n* = 3). In these experiments cytochrome *c* oxidase activity was 32.6 K min$^{-1}$ $10^{-9}$ cells in Ara-C treated cells *vs* 21.3 K min$^{-1}$ $10^{-9}$ cells in controls. The enhanced cytochrome *c* oxidase activity in Ara-C treated Molt 4 cells is accompanied by a higher oxygen consumption due to an enhanced mt respiration.

*Synchronisation of the cells*

The $G_1$ phase cell fraction used as a starting population for synchronised cell cycle progression was at least 95% pure. Upon reculturing the control cells reached the S phase at 9 h. Fourteen per cent of the cells had entered the $G_2M$ phase after approximately 10 h (Figure 1).

When $G_1$ cells were cultured in the presence of Ara-C (0.05 μM) they did not exhibit an increase of cellular DNA and cell numbers remained constant.

*Effects of Ara-C upon mt activity in $G_1$ phase cells*

*Total protein and cell size* The protein content of the cells gradually rose from approximately 100 mg $10^{-9}$ cells at t = 0 to 120 mg $10^{-9}$ cells at t = 9 h. Until then no differences were found between cellular protein in control cells and cells cultured in the presence of Ara-C (Figure 2a). Beyond 9 h the protein level in cells exposed to Ara-C continued to increase, whereas in control cells no further increase was demonstrated. The cell size as measured by flowcytometry appeared similar in Ara-C treated cells and controls throughout the 12 h of follow-up (Figure 2b).

*Cytochrome* c *oxidase activity* $G_1$ cells in the starting population (t = 0) showed a cytochrome *c* oxidase activity of 8.4 ± 1.1 K min$^{-1}$ $10^{-9}$ cells. As control $G_1$ cells progressed through the cell-cycle, the activity of cytochrome *c* oxidase increased to a maximum value of approximately 17.5 K min$^{-1}$ $10^{-9}$ cells at 9 h of culturing, when the majority of the cells had arrived in S phase. Further, in the course of the cell cycle, cytochrome *c* oxidase activity remained more or less constant (Figure 3a). Cytochrome *c* oxidase activity of $G_1$ cells cultured in the presence of Ara-C continued to increase to a value of 26 K min$^{-1}$ $10^{-9}$ cells after 12 h. Moreover the increase started at an earlier point in time and proceeded at a higher rate in the Ara-C treated cells (Figure 3a). The specific cytochrome *c* oxidase activity (activity of cytochrome *c* oxidase per mg protein) was also higher in the Ara-C exposed $G_1$ cells compared with control cells (Figure 3b). In the presence of both Ara-C and doxycycline, a specific inhibitor of mt protein synthesis (Kroon & Van den Bogert, 1983), cell cycle progression was inhibited to the same as in the presence of Ara-C alone (data not shown), but under these circumstances Ara-C did not enhance cytochrome *c* oxidase activity (Figure 3a). The activity of the enzyme remained at the level of $G_1$ cells in the starting population. The addition of doxycycline had no influence on the increase of the cellular protein content and the size of the cells during the 12 h of follow-up (Figure 2a,b).

**Figure 2** $G_1$ cells, separated by counterflow centrifugation, were recultured in complete medium as controls (○), in the presence of Ara-C (0.05 μM) (●) and with both Ara-C (0.05 μM) and doxycycline (10 μg ml$^{-1}$) (★). Protein content, **a**, and cell size, **b**, were assessed at various time points after reculturing. Protein content is expressed in mg $10^{-9}$ cells, cell size (RFS) in arbitrary units. A representative experiment is shown.

**Figure 1** $G_1$ Molt 4 cells, separated by counterflow centrifugation, were recultured with or without Ara-C (0.05 μM) for up to 12 h. Series of DNA histograms in time show the progression of control $G_1$ cells through the cell cycle (left); on the right side the DNA histograms at similar timepoints, when $G_1$ cells were cultured in the presence of Ara-C, are shown. Each DNA histogram is graphically normalised to equal peak height. Abscissa: relative amount of cellular DNA; z-axis: subsequent time points; ordinate: relative cell numbers.

**Figure 3** $G_1$ cells were separated by counterflow centrifugation and regrown in complete medium in the presence of 0.05 μM Ara-C (closed symbols), in the presence of both 0.05 μM Ara-C and 10 μg $ml^{-1}$ doxycycline (★), or as controls (open symbols). The activities of mt enzymes were measured at subsequent time points during reculturing. **a**, activities of cytochrome *c* oxidase on a per cell base, expressed as absolute values (K $min^{-1}$ $10^{-9}$ cells). The data from two independent experiments is shown. **b**, enzyme activities related to the total cellular protein content and expressed as specific activities: cytochrome *c* oxidase (○, ●) (K $min^{-1}$ $mg^{-1}$ protein) and citrate synthase (□,■) (μmol $min^{-1}$ $mg^{-1}$ protein). The data from a representative example is shown.

**Figure 4** $G_1$ cells were separated by counterflow centrifugation and recultured in complete medium without Ara-C (○) or with 0.05 μM Ara-C (●). The concentrations of ATP **a** and ADP **b** in the cells were determined at various time points; the values are expressed in nmol $10^{-6}$ cells. A representative example is shown.

*Citrate synthase activity* Citrate synthase activity in Ara-C treated cells both expressed per $10^9$ cells and per mg protein (specific enzyme activity) did not rise above the activity in control cells during the 12 h of exposure of $G_1$ cells (Figure 3b).

*ATP and ADP pools* The level of ATP in control cells increased throughout the $G_1$ into the S phase from 3.4 to 6.3 nmol $10^{-6}$ cells. During the first 6 h of culturing the ATP levels in Ara-C exposed $G_1$ phase cells and in control $G_1$ phase cells increased at a comparable rate. From then onward ATP levels of control cells declined, whereas in the presence of Ara-C a further rise was observed till approximately 7.2 nmol $10^{-6}$ cells (Figure 4a). The ADP content of control and Ara-C exposed cells slowly increased from 0.15 nmol $10^{-6}$ cells at t = 0 to 0.35 nmol $l^{-6}$ cells at t = 10.5 h. For at least 10 h ADP pools were comparable in the two groups (Figure 4b).

*Uptake of a mt specific dye* The increase in the capacity of the cells to accumulate mt specific dye during the first 6 h of Ara-C exposure was similar to that in control $G_1$ cells. Beyond this time less dye was found to accumulate in control cells, whereas Ara-C treated cells demonstrated a further increase (Figure 5).

*Levels of transcripts of the genes for subunit II (COX II) and IV (COX IV) of cytochrome c oxidase* The amount of COX II trancripts per μg toal cellular RNA was higher in the Ara-C treated $G_1$ cells when compared with control $G_1$ cells. Already 45 min after exposure to Ara-C the amount of COX II transcript was increased relative to that in control cells (Figure 6, upper part). The concentration of the nuclear-DNA encoded COX IV transcript per μg total cellular RNA was similar in Ara-C treated cells and controls (Figure 6, lower part). The amount of mt-DNA remained similar in Ara-C treated cells and controls (data not shown).

### Discussion

$G_1$ phase cells exposed to Ara-C at a concentration inhibitory to cell proliferation, shown an increased respiration rate and an enhanced activity of cytochrome *c* oxidase. Moreover Ara-C treated $G_1$ cells display a higher capacity to accumulate the mt specific dye Di-O-C(5)3. This suggests that these $G_1$ cells possess a larger mass of mt membranes and an

**Figure 5** $G_1$ cells were separated by counterflow centrifugation and recultured in complete medium in the presence of 0.05 $\mu$M Ara-C (●) or without Ara-C (○). The capacity of the cells to accumulate mt specific dye Di-O-C(5)3 was assessd at various points in the course of time.

**Figure 6** $G_1$ phase cells were obtained by counterflow centrifugation and recultured in complete medium in the presence of 0.05 $\mu$M Ara-C, or as controls. At different times of culturing total RNA was extracted, separated, blotted (3 $\mu$g per slot) and hybridised to probes for the COX II mRNA (upper part) and for the COX II mRNA (lower part). One and the same filter was used for hybridisation with the COX II probe and, after dehybridisation, for rehybridisation with the COX IV probe. To arrive at comparable signals for both the COX II and the COX IV mRNAs, an exposure time of 1 h was required for the COX II probe and of 20 h for the COX IV probe.

increased total mt membrane potential and synthesise an increased amount of ATP. The discrepancy in time between the elevations of cytochrome *c* oxidase activity and the concentration of ATP may point at an enhanced turnover of ATP during the early hours of Ara-Ç exposure.

Studies on the effect of specific inhibition of mt protein synthesis and measurement of the concentration of the transcripts of the mt encoded subunit of cytochrome *c* oxidase, COX II, suggest that Ara-C induces cytochrome *c* oxidase synthesis by an enhanced transcription and translation of the mt genome. Since the mt genome is transcribed in total it is likely that the synthesis of other polypeptides which are encoded on mt-DNA, such as subunits of ATP synthase, is stimulated as well. An observation in favour of this supposition is the enhanced respiratory rate in Ara-C treated cells.

Control $G_1$ phase cells progress into the S phase while Ara-C treated cells do not (Figure 1). However a number of arguments favour the supposition that the enhanced mt acivities observed are induced by Ara-C and are not secondary to perturbation of the cell cycle.

(1) Counterflow centrifugation provides an almost pure suspension of viable $G_1$ phase cells, which also maintain a high degree of synchronisation upon reculturing (Vierwinden *et al.*, 1982). Enhancement of cytochrome *c* oxidase activity was observed already after exposure to Ara-C for only 1.5 and 3 h, while it took 9 h of reculturing, for the majority of control $G_1$ phase cells to enter the S phase of the cell cycle.

(2) The few cells which entered S earlier than the majority of the $G_1$ cells, could hypothetically be responsible for a depression of cytochrome *c* oxidase activity in the control sample. This is unlikely however, because it has been demonstrated that the cytochrome *c* oxidase activity in untreated Molt 4 cells increases during $G_1$ and remains at a plateau level during the S phase of the cell cycle (Van den Bogert *et al.*, 1988).

The generally accepted mechanisms of Ara-C cytotoxicity implying inhibition of nuclear-DNA polymerase and incorporation of the phosphorylated metabolite into nuclear-DNA (Cozzarelli, 1977) can thus not acocunt for the observed effects in early $G_1$ phase cells.

The observation that an increased amount of functioning cytochrome *c* oxidase coexists with an enhanced concentration of the mRNAs of only those subunits which are encoded on the mt genome (such as COX II) could indicate that there is a surplus of nuclear-DNA encoded subunits (such as COX IV), as was also suggested by Schatz (1968) and by Weiss and Kolb (1979) and may suggest a regulatory role of the mt genome products (Van den Bogert *et al.*, 1988). It must be stressed here, that the much higher copy number of mt mRNAs per cell (such as COX II mRNA) in comparison with that of the nuclear mRNAs for mt proteins (such as COX IV mRNA) does not necessarily mean that mt mRNAs are functionally in excess over the nuclear messengers (De Vries *et al.*, 1990). The very particular properties of mt mRNAs (lacking ribosome-binding sites and leader sequences), mt tRNAs (lacking loops that are essential in all other systems) and mt ribosomes (having very small rRNAs and a very high protein content) may well lead to a very slow or inefficient translation process that is only able to function when mRNA levels are high. Dysregulation in time or intensity of the expression of the mt genome is therefore even more likely to interfere with the function and survival of the cell. Enhancement by Ara-C of the expression of mt-DNA in $G_1$ phase cells, implying such a dysregulation, could partly explain earlier data suggesting a cytotoxic effect of Ara-C exerted on non-S phase cells (Haanen *et al.*, 1985; Valeriote, 1982).

If our data imply a type of unbalanced growth it indicates that increased transcription and translation of mt-DNA must be a very early or even initiating event. $G_1$ cells which were inhibited by Ara-C in fact continued to increase in size and outgrew control cells, be it measurable only after 10–12 h (Figure 2). Our data is not in conflict with inhibition of proliferation by an increased expression of differentiation antigens. The phenomenon of cell differentiation is accompanied by an enhancement of mt activity and mt mass (Pederson, 1978). Craig *et al.* (1984) provided strong evidence that Ara-C induces differentiation in non-S phase cells of a human leukaemic cell line. Reports in the literature on differentiation induction by Ara-C are still conflicting (Sachs, 1978; Reiss *et al.*, 1986).

We demonstrated that dysregulation in time and intensity of the expression of the mt genome resulting in enhanced oxidative phosphorylation occurred during the $G_1$ phase of the cell cycle and at Ara-C concentrations, which inhibit nuclear-DNA synthesis and cell proliferation. The present study does not allow conclusions about a causal relationship between the effect of mt activities and the strong antileukaemic effect of the drug. However, mt activity may be an important target in attaining cytostasis (Van den Bogert *et al.*, 1986) or even cytotoxicity in $G_1$ phase cells and deserves further investigation.

This work was supported by the Netherlands National Cancer Foundation, by the Ank van Vlissingen Foundation and by the Upjohn Company.

### References

ANDERSON, S., BANKIER, A.T., BARRELL, B.G. & 11 others (1981). Sequence and organization of the human mitochondrial genome. *Nature*, **290**, 457.

BIRNBOIM, H.C. (1988). Rapid extraction of high molecular weight RNA from cultured cells and granulocytes for Northern analysis. *Nucleic Acids Res.*, **16**, 1487.

BORST, P., RUTTENBERG, G.J.C.M. & KROON, A.M. (1967). Mitochondrial DNA I. Preparation and properties of mitochondrial-DNA from chick liver. *Biochim. Biophys. Acta*, **149**, 140.

CHURCH, G.M. & GILBERT, W. (1984). Genetic sequencing. *Proc. Natl Acad. Sci., USA*, **81**, 1991.

COZZARELLI, N.R. (1977). The mechanism of action of inhibitors of DNA synthesis. *Ann. Rev. Biochem.*, **46**, 641.

CRAIG, R.W., FRANKFURT, O.S., SAKAGAMI, H., TAKEDA, K. & BLOCH, A. (1984). Macromolecular and cell cycle effects of different classes of agents inducing the maturation of human myeloblastic leukemia (ML-1) cells. *Cancer Res.*, **44**, 2421.

DENHARDT, D.T., EDWARDS, D.R. & PARFETT, C.L.J. (1986). Gene expression during the mammalian cell cycle. *Biochim. Biophys. Acta*, **865**, 83.

DE VRIES, H., HOLTROP, M., MUUS, P., PENNINGS, A., DE JONGE, J. & VAN DEN BOGERT, C. (1990). Expression of genes for mitochondrial proteins during proliferation of human cells. In *Structure, Function and Biogenesis of Energy Transfer Systems*. Quagliariello, E., Papa, S., Palmieri, F. & Saccone, C. (eds) p. 127. Elsevier: Amsterdam.

DE VRIES, H. & VAN'T SANT, P. (1983). Interplay between different genetic systems in eukaryotic cells: nucleocytoplasmatic, mitochondrial interrelations. In *Horizons in Biochemistry and Biophysics, 7- Genes: Structure and expression*, Kroon, A.M. (ed.) p. 249. John Wiley and Sons: Chichester - New York - Brisbane - Toronto - Singapore.

DE WITTE, T., PLAS, A., KOEKMAN, E. & 4 others (1984). Separation of human bone marrow by counterflow centrifugation monitored by DNA-flowcytometry. *Br. J. Haematol.*, **58**, 249.

FEINBERG, A.P. & VOGELSTEIN, B. (1983). A technique for radiolabeling DNA restriction endonuclease fragments to high specific activity. *Anal. Biochem.*, **132**, 6.

GOHDE, W. (1973). In *Zell Zyklus Analysen mit dem Impuls-cytophotometer*. Westfälischen Wilhelms Universität, Münster, Germany, Thesis.

GRANGER, D.L. & LEHNINGER, A.L. (1982). Sites of inhibition of mitochondrial electron transport in macrophage-injured neoplastic cells. *J. Cell Biol.*, **95**, 527.

HAANEN, C., MUUS, P., RAIJMAKERS, R., DRENTHE-SCHONK, A., SALDEN, M. & WESSELS, J. (1985). Studies on the cytotoxicity of cytosine arabinoside. *Semin. Oncol.*, **12**, 120 (suppl. 3).

HAANEN, C., MUUS, P. & PENNINGS, A. (1986). The effect of cytosine arabinoside upon mitochondrial staining kinetics in human hematopoietic cells. *Histochemistry*, **85**, 609.

JOHNSON, L.V., WALSH, M.L., BOCKUS, B.J. & CHEN, L.B. (1981). Monitoring of relative mitochondrial membrane potential in living cells by fluorescence microscopy. *J. Cell Biol.*, **88**, 526.

KUFE, D.W., MAJOR, P.P., EGAN, E.M. & BEARDSLEY, G.P. (1980). Correlation of cytotoxicity with incorporation of Ara-C into DNA. *J. Biol. Chem.*, **255**, 8997.

KRISHAN, A. (1975). Rapid flow cytofluorometric analysis of mammalian cell-cycle by propidium iodide staining. *J. Cell Biol.*, **66**, 188.

KROON, A.M. & VAN DEN BOGERT, C. (1983). Antibacterial drugs and their interference with the biogenesis of mitochondria in animal and human cells. *Pharmac. Weekbl. (Sci.)*, **5**, 81.

KROON, A.M. & VAN DEN BOGERT, C. (1987). Biogenesis of mitochondria and genetics of mitochondrial defects. *J. Inher. Metab. Dis.*, **10**, 54 (suppl. 1).

MARIOTTINI, P., CHOMYN, A., RILEY, M., COTTRELL, B., DOOLITTLE, R.F. & ATTARDI, G. (1986). Identification of the polypeptides encoded in the unassigned reading frames 2, 4, 4L and 5 of human mitochondrial-DNA. *Proc. Natl Acad. Sci. USA*, **83**, 1563.

MUUS, P., HAANEN, C., PENNINGS, A., RUITENBEEK, W. & VAN DEN BOGERT, C. (1987). Influence of cytarabine on mitochondrial function and mitochondrial biogenesis. *Semin. Oncol.*, **14**, 245 (suppl. 1).

PEDERSON, P.L. (1978). Tumor mitochondria and bioenergetics of cancer cells. *Prog. Exp. Tumor Res.*, **22**, 190.

PETERSON, G.L. (1977). A simplification of the protein assay method of Lowry *et al.*, which is more generally applicable. *Anal. Biochem.*, **83**, 346.

PLAS, A., DE WITTE, T., WESSELS, J. & HAANEN, C. (1988). A new multichamber counterflow centrifugation rotor with high separation capacity and versatile potentials. *Exp. Hematol.*, **16**, 355.

REISS, M., GAMBA-VITALO, C. & SARTORELLI, A.C. (1986). Induction of tumor cell differentiation as a therapeutic approach: preclinical models for hematopoietic and solid neoplasms. *Cancer Treat. Rep.*, **70**, 201.

SACH, L. (1978). The differentiation of myeloid leukemia cells: new possibilities for therapy. *Br. J. Haem.*, **40**, 509.

SCHATZ, G. (1968). Impaired binding of mitochondrial adenosine triphosphate in the cytoplasmic 'petite' mutant of Saccharomyces cerevisia. *J. Biol. Chem.*, **243**, 2192.

SCHATZ, G. & BUTOW, R.A. (1983). How are proteins imported into mitochondria? *Cell*, **32**, 316.

SOLOMONS, C.C., TAN, S. & ALDRETE, J.A. (1977). Platelet metabolism and malignant hyperthermia. In *Malignant Hyperthermia*, Eldrete, J.A., Britt, B.A. (eds) p. 221. Grune and Stratton: New York, USA.

SRERE, P.A. (1969). Citrate synthase. In *Methods in Enzymology*, 13, Löwestein, J.M. (ed.) p. 3. Academic Press: London.

VALERIOTE, F. (1982). Cellular aspects of the action of cytosine arabinoside. *Med. Pediatr. Oncol.*, **1**, 5 (suppl. 1).

VAN DEN BOGERT, C., VAN KERNEBEEK, G., DE LEIJ, L. & KROON, A.M. (1986). Inhibition of mitochondrial protein synthesis leads to proliferation arrest in the $G_1$ phase of the cell cycle. *Cancer Lett.*, **32**, 41.

VAN DEN BOGERT, C., MUUS, P., HAANEN, C., PENNINGS, A., MELIS, T.E. & KROON, A.M. (1988). Mitochondrial biogenesis and mitochondrial activity during the progression of the cell cycle of human leukaemic cells. *Exp. Cell Res.*, **178**, 143.

VAN EGMOND, J. & HILLEN, H.F. (1978). Evidence for a slow onset of DNA-synthesis in bone-marrow derived from computer analysis of pulse-cytophotometry DNA-histograms. In *Pulse-Cytophotometry (part III)*, 3rd Int. Symp. Vienna, Lutz, D. (ed.) p. 117. European Press: Gent, Belgium.

VIERWINDEN, G., DRENTHE-SCHONK, A.M., PLAS, A.M. & 6 others (1982). Variations of the phosphorylation of 1-β-D arabinofuranuosyl cytosine in human myeloid leukaemic cells related to the cell cycle. *Leuk. Res.*, **6**, 251.

WEISS, H. & KOLB, H.J. (1979). Isolation of mitochondrial succinate: ubiquinone reductase and cytochrome *c* oxidase from Neurospora crassa using nonionic detergent. *Eur. J. Biochem.*, **99**, 139.

WHITE, T.W. & BOHMAN, R. (1981). Proliferation of mitochondria during the cell cycle of the human cell line. *J. Cell. Biol.*, **89**, 256.

WOODCOCK, D.M. (1987). Cytosine arabinoside toxicity: molecular events, biological consequences and their implications. *Semin. Oncol.*, **14**, 251 (suppl. 1).